# A Failure to Communicate? How Public Messaging Has Strained the COVID-19 Response in the United States

**DOI:** 10.1089/hs.2020.0190

**Published:** 2021-02-18

**Authors:** Molly A. Sauer, Shaun Truelove, Amelia K. Gerste, Rupali J. Limaye

**Affiliations:** Molly A. Sauer, MPH, is a Research Associate; Shaun Truelove, PhD, is an Assistant Scientist; Amelia K. Gerste, MSPH, is a Communications Specialist; and Rupali J. Limaye, PhD, MPH, MA, is an Associate Scientist and Director of Behavioral and Implementation Science (IVAC); all in the Department of International Health and the International Vaccine Access Center, Johns Hopkins Bloomberg School of Public Health, Baltimore, MD. Shaun Truelove is also an Assistant Scientist, Department of Epidemiology, and Rupali J. Limaye is also an Associate Scientist, Department of Epidemiology and Department of Health, Behavior, and Society; all at the Johns Hopkins Bloomberg School of Public Health, Baltimore, MD.

**Keywords:** COVID-19, Risk communication, Public health preparedness/response, Pandemic response, Infectious diseases

## Abstract

A pandemic, especially when caused by a novel virus, induces tremendous uncertainty, fear, and anxiety. To mitigate panic and encourage appropriate behavioral action, communication is critical. The US Centers for Disease Control and Prevention's Crisis and Emergency Risk Communication (CERC) guidance is designed to assist public health authorities, government officials, and other stakeholders in using risk communication during an emergency. For each of the 6 core communication principles outlined in the CERC guidance, we describe the use or nonuse of these principles at critical points during the coronavirus disease 2019 (COVID-19) pandemic by US public health and government officials. With the knowledge that the pandemic will continue to rage for some time and that new communication challenges will arise, including issues related to vaccination and treatment options, many lessons are to be learned and shared. To reduce fear and uncertainty among those living in the United States, COVID-19 communication should be rapid and accurate, while building credibility and trust and showcasing empathy—all with a unified voice.

On december 31, 2019, China reported a cluster of pneumonia cases of unknown cause that would later be identified as severe acute respiratory syndrome coronavirus 2 (SARS-CoV-2).^1^ Recognizing its widespread transmission, the World Health Organization (WHO) declared novel coronavirus disease 2019 (2019-nCoV, later called COVID-19) a public health emergency of international concern on January 30, 2020,^2^ and a pandemic on March 11, 2020.^3^ Soon after, COVID-19 was declared a national emergency in the United States, resulting in a number of control measures: social distancing; school, bar, cinema, and restaurant closures; cancellation of large gatherings; and a transition to remote work for many businesses^4^ (see Figure 1 for a timeline of major pandemic-related global and US events). Public health scientists were suddenly at the forefront of an unprecedented crisis, tasked with taming a quickly spreading novel virus.^5^ More than 10 months later, a safe and effective vaccine is only just becoming available, over 430,000 people have died in the United States,^6^ millions have lost jobs, and a majority of Americans now live their lives virtually.

**Figure 1.**
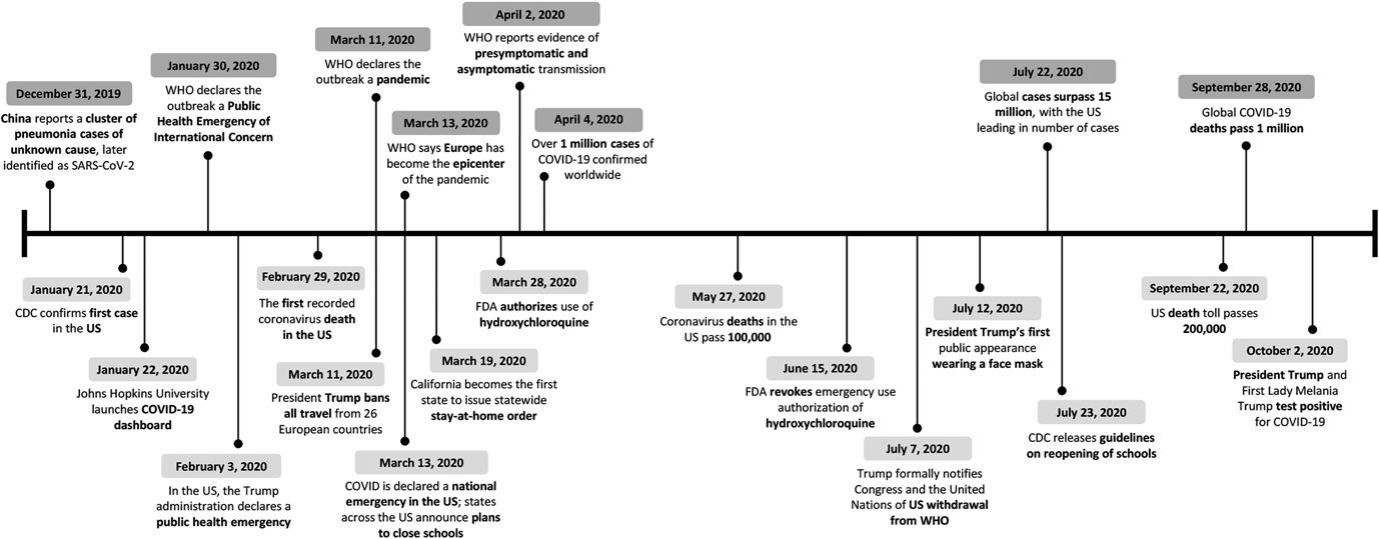
Timeline of major pandemic-related global and US events. Abbreviations: CDC, Centers for Disease Control and Prevention; COVID-19, coronavirus disease 2019; FDA, US Food and Drug Administration; SARS-CoV-2, severe acute respiratory syndrome coronavirus 2; US, United States; WHO, World Health Organization.

These widespread public health measures have been accompanied by a massive flow of COVID-19 information, misinformation, and disinformation. We are concurrently inundated with a global epidemic of misinformation, or an infodemic, primarily being spread through social media platforms; its effects on public health cannot be underestimated.^7^ Thus, the pandemic provides an opportunity to develop infodemic management approaches.^8^

In 2002, the US Centers for Disease Control and Prevention (CDC) published the Crisis and Emergency Risk Communication (CERC) manual to inform successful public, partner, and stakeholder communication during crises and emergencies; this manual was updated in 2012, 2014, and 2018.^9,10^ The CERC manual integrates elements from risk, crisis, and health communication theories and outlines 6 core communication principles: (1) be first, (2) be right, (3) be credible, (4) express empathy, (5) promote action, and (6) show respect (Figure 2). It posits that trust, adherence to public health recommendations, and support during an emergency can be achieved, partly by providing information to enable the public to make sense of the emergency.^11,12^ Many of these principles are intertwined and designed to be employed together; as such, failure to adhere to even 1 principle affects application of the broader framework.

**Figure 2.**
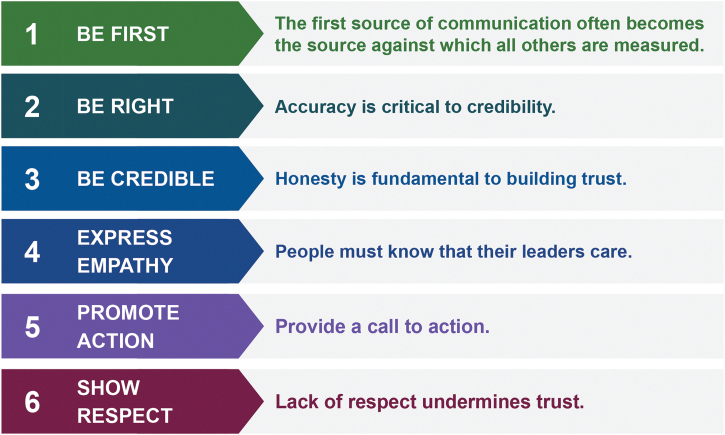
Crisis and Emergency Risk Communication framework principles. Adapted from the US Center for Disease Control and Prevention *Crisis & Emergency Risk Communication (CERC) Manual*.^10^

The novelty of this virus and the constantly changing science around it has led to many communication challenges. Because of the uncertainty around risks related to COVID-19, traditional and social media have inadvertently induced panic and feelings of fear, sadness, and anger.^13^ Carefully planned crisis communication plays a critical role in preventing and mitigating pandemics, by alleviating fear and anxiety and supporting adherence to public health recommendations.^14^ The information environment around this pandemic highlights the importance of effective communication. A rise in conspiracy theories, fake news, and misinformation has made it increasingly challenging for the public to distinguish scientific evidence from misinformation.^15^

Risk communication—“the exchange of information among interested parties about the nature, magnitude, significance, or control of a risk”^16^—has a long history of research and practice that informs many public health campaigns.^17^ At its core, risk communication focuses on timely, accurate, effective dissemination of high- or low-hazard information to at-risk populations, aiming to minimize the gap between knowledge and action.^16^ It also includes communication and advice to the public on behaviors to proactively cope with risk.^18^ The risks associated with miscommunication during the COVID-19 pandemic are tremendous, especially because of declining trust in and credibility of authorities and governments.^16^

Effective mobilization in response to a crisis like COVID-19 starts with national leadership.^19^ Because of their infrequent nature, emergencies create high levels of uncertainty and an unstable environment.^13,20^ People need information on basic needs, like food, water, and shelter, as well as guidance on how to protect their health and safety and limit morbidity and mortality.^21^ Accuracy and timeliness are hallmarks of effective emergency communication.^22,23^ Effective communication from leadership is crucial in building trust in the response and ensuring compliance with key public health measures; failure to do so can have lasting, far-reaching consequences.

In this article, we apply the CERC framework to examine the COVID-19 response in the United States. We provide illustrative examples as to how each of the 6 core principles were and were not used by various public health authorities and government leaders in the first 10 months of the COVID-19 pandemic, through October 2020, and the consequences of those successful or failed applications. Through this exercise, we aim to rapidly identify and describe lessons learned to strengthen both ongoing and planned COVID-19 communication efforts—particularly in the midst of the fall and winter surge—and future infectious disease epidemics demanding effective crisis and emergency risk communication.

## Applying CERC Principles in the United States

### Be First

Early communication about an emerging crisis and the necessary actions for the public—even if details are limited or likely to change—is vital to setting the stage for public trust. CERC highlights that the first source of information frequently becomes the benchmark for future communication; as a crisis evolves and new information emerges, the early voices are often more trusted. We sought to characterize communication from several of these first sources of information.

Failure to “be first” can have lasting consequences. Since COVID-19 was first detected in the United States in January 2020,^24^ the federal government's public communication related to the virus has lagged, undermining public trust in the response and elevating the risk to public health.^10,25^ More than 2 months after the first reported US cases, officials at the highest levels continued to downplay the urgency and severity of the pandemic and stall on a national response or clear public guidelines to mitigate risk.^26-29^ This lack of transparency, coupled with a delayed government response and inconsistent messaging, continued for months and generated even greater uncertainty among the public—individuals instead sought out information from a range of sources, many of which were not grounded in science.^30-32^ In addition to failed risk communication, the delayed federal response and stream of misinformation crippled subnational response capabilities and put federal and state leadership at odds.

Conversely, early action from 2 institutions established themselves as trusted sources of data. These institutions were the Johns Hopkins University Center for Systems Science and Engineering, which created the first comprehensive COVID-19 dashboard,^6,33^ and the Institute for Health Metrics and Evaluation at the University of Washington, which released the first long-range projections of global COVID-19 morbidity and mortality under various response scenarios.^34^ The publicly accessible COVID-19 dashboard continues to provide reliable real-time data on cases, deaths, and other key indicators at the national and subnational levels. Even now, almost 1 year since the earliest reported cases in China, these resources continue to be the primary and preferred information sources for governments, media, and public health institutions. Being the first to generate comprehensive COVID-19 data was key to establishing trust and conferring resilience, which is especially important because projections are notoriously dynamic. That trust and credibility has been maintained and strengthened by continuing to provide consistent, robust analysis even as some authorities use alternate data and calculations that may downplay the pandemic's severity in their regions.^35-37^

### Be Right

Accuracy and transparency are crucial to building and maintaining credibility; the COVID-19 response has revealed critical failures in this area. Particularly when facing a novel threat with many uncertainties, it is essential that authorities communicate what is currently known, what is unknown, and what steps are being taken to fill those gaps.^38^ CERC advises leaders to acknowledge areas where they do not yet have information and to articulate the work being done to gather evidence. Contradictory messages from political and scientific leadership can greatly undermine the public's trust and generate misinformation.

Shifting guidelines on mask use demonstrate the challenges of managing unknowns and the importance of upholding the “be right” principle.^39^ At the outset of the pandemic, WHO and US public health authorities advised the public not to wear masks, despite limited evidence on their potential benefit or detriment to disease control.^40^ On February 29, 2020, US Surgeon General Dr. Jerome Adams communicated that masks were not effective in preventing COVID-19 infection among the general public.^41^ The following week, Dr. Anthony Fauci echoed the statement that face masks were not necessary for the general public and would not offer the level of protection people perceived.^42^ At the time, studies were underway to assess whether masks—particularly cloth masks, to reduce demand for surgical and N95 masks needed by healthcare workers—could help reduce transmission,^43,44^ especially as more evidence emerged about asymptomatic cases. Less than a month later, the CDC issued new guidance advising the public to wear cloth masks to help reduce transmission. Both Dr. Adams and Dr. Fauci acknowledged the emerging evidence about transmission and the importance of masks and changed their messages, advising individuals to wear face coverings while in public.^45,46^

While likely uncomfortable for many leaders, communicating uncertainties and emphasizing the work being done to address them can help strengthen public support and compliance.^32,47^ Identifying and investigating gaps in our understanding is core to the scientific process, and this should be communicated clearly. People may be more receptive to unknowns when they are presented as steps in the robust scientific process rather than the result of failures by authorities.^32,48^

### Be Credible

A novel pathogen presents a complex, dynamic situation for public health officials and health communicators. Information shared one day may be completely reversed the next as more evidence emerges; guidance can quickly become obsolete. The public can often be forgiving of perceived errors from authority figures. Maintaining credibility is driven heavily by not only being at the forefront and being correct, but also being honest and transparent, even when sharing difficult information. CERC emphasizes the importance of transparency and not shielding the public from information because of fears of panic or embarrassment.^11,17,38^

President Trump declared COVID-19 an emergency in the United States on March 13, 2020. In September, it came to light that he downplayed the threat of the virus in a March interview, and he said he did so because he was worried about creating panic.^49^ A week after the March interview, at a White House press briefing, he asserted that the number of cases would be close to zero in a few days.^50^ Experts suggested he had another option: calmly and accurately communicate to the American people about the risks related to the virus and preventive actions they could take to reduce their risk. When asked if he perhaps misled the public with these comments, he argued that he did so in order to reduce panic.^51^ He also asserted that the virus would disappear on its own, even as scientists argued that the pandemic would not be eradicated without widespread distribution of a COVID-19 vaccine.^52^ Withholding information from the public and communicating known falsehoods undermined President Trump's credibility and damaged public trust.

As a leading health authority in the United States and globally, the CDC is seen as one of the most scientifically rigorous and trusted sources for health and disease guidance. With schools closed since March, school officials looked to the CDC for directions on reopening and in-person learning. When this guidance finally came in late July, only weeks before many schools would normally start, it was flagged as “very tough and expensive”^53^ by President Trump and others. Two weeks later, the CDC issued new guidance that was notably less strict than the original recommendations.^53^ This perceived bowing to political pressure, rather than relying on scientific rigor, was another blow to the CDC's credibility.^54^

Ambiguous or contradictory guidance amplifies already stressful crises and increases risk. South Dakota Governor Kristi Noem described critical protective actions like wearing masks as optional at a large Fourth of July event and specifically stated social distancing would not be in place.^55^ This contradicted public health guidance and placed the onus on individuals to determine their own risk.

Trust is a critical antecedent to government legitimacy and credibility, which shapes citizens' responses to government demands.^56^ Residents who perceive their public leaders as trustworthy are more likely to comply with government demands; this is particularly relevant to the COVID-19 crisis because an effective response depends on collective compliance of public health guidelines.^57^

### Express Empathy

Crises inject a great deal of uncertainty, fear, and anxiety into people's lives. Acknowledging these feelings can help build trust, calm anxiety, and restore order. The CERC manual suggests that empathy should be expressed early on in any messaging, because empathy is critical to create rapport. However, empathetic communication alone is insufficient. Authorities who publicly acknowledge the shared sacrifice and anxiety experienced by their communities but privately fail to abide by the same standards damage public trust and work against risk communication initiatives. Throughout the COVID-19 pandemic, the American people have been shown the full spectrum from apathy to empathy, and in some cases the empathy shown has been called into question by subsequent personal actions.

Political and public health leaders have drawn on personal experience with the disease—their own illness or that of loved ones, and the experiences of family and friends involved in caring for COVID-19 patients—and the challenges of restrictions on gathering, school closures, and other interventions, particularly leading up to the holiday season. Unfortunately, for every expression of empathy, we can find an example of indifference toward the struggling American public or a failure to follow through with appropriate actions. In March, Texas Lieutenant Governor Dan Patrick was criticized for asserting “there are more important things than living” after suggesting that senior citizens might be willing to die to save the economy and that some people need to take some risks to get the country back to normal.^58^ On school reopening, US Secretary of Education Betsy DeVos forcefully pushed for an accelerated school reopening, despite withholding necessary funding for safe operation as outlined by CDC recommendations. DeVos downplayed the risk of transmission and threatened to take away funding from schools that did not reopen.^59^ These statements trivialize the losses and struggles that so many Americans have experienced.

In other cases, leaders rallied the public around a shared experience and communicated the need for collective sacrifice to break the cycle of transmission, expressing empathy with their constituents in this challenging time. However, their empathetic communication was contradicted by their actions. California Governor Gavin Newsom attended a large party shortly after communicating to California residents the need to avoid large gatherings, even as many families had hoped to do so around the holidays.^60^ Denver Mayor Michael B. Hancock urged the public to avoid traveling for Thanksgiving, empathizing that many people would miss time with family as a result, and then flew across the country for his own personal holiday travel.^61^ Both officials acknowledged the contradiction and failure to abide by the same expectations they had communicated to the public, but their actions damaged credibility and reinforced public perceptions that political elites were held to different standards than the general public with whom they claimed to empathize.

### Promote Action

In addition to providing information, crisis communication must include clear, concise, and concrete actions for the public. Emotions run high during emergencies, particularly when facing new threats, and offering people meaningful steps to take may help provide a sense of control in protecting themselves and others.^10,25^ CERC highlights the importance of these clear, concise messages about what actions should be taken.^25^ In some situations, offering a range of actions may be most impactful: maximum, moderate, and minimum responses.^62^ Public health messaging during this pandemic has included full stay-at-home orders (maximum); guidance to wear masks, avoid public transit and crowds, and practice social distancing and hand hygiene (moderate); and if those are not all feasible, to at least practice social distancing and hand hygiene (minimum). Critically, communications to promote actions should highlight their benefits and importance.

Several state and local leaders demonstrated effective application of this principle. Maryland Governor Larry Hogan and Baltimore City Mayor Bernard C. “Jack” Young convened weekly press conferences that consistently included specific, simple actions the public should take to protect themselves and help mitigate the virus's spread. In July, as he expanded the state's mask order, Governor Hogan empathized with residents of Maryland while emphasizing the importance of wearing masks and the science behind the order.^63^ Clear messages with a simple, concrete action can help empower individuals to protect themselves and provide a sense of control.^25^ Unified, apolitical messaging around these actions is also crucial to avoid actions taking on a partisan spin. Early in the pandemic, Republican governors in generally “blue states” (eg, Governor Hogan and Governor Phil Scott of Vermont) demonstrated the importance of a collective crisis response that bridged the political aisle, noting, for example, that wearing masks should not be a political statement.^63,64^

### Show Respect

With the high level of uncertainty in an emergency context, respectful communication is paramount. With many unknowns about COVID-19, including its long-term effects and what comorbid conditions pose the greatest risk, the public has continuously experienced high levels of uncertainty about prevention, treatment, and care, which have been amplified as vaccine candidates and potential rollout strategies have been announced. Respectful communication promotes cooperation and rapport and engages and values community inputs, all of which are essential to promote adherence to public health recommendations.

In late 2020, as 3 COVID-19 vaccines released Phase 3 clinical trial data demonstrating safety and high efficacy, authorities moved forward with planning for distribution. State and federal leaders were tasked with communicating to the public about the phases of vaccine availability, which prioritized healthcare workers, long-term care residents, other frontline and essential workers, and individuals at highest risk of infection and severe illness. However, many of these communities have experienced historical trauma associated with public health and medical interventions and have also faced disproportionate health and economic impacts from current COVID-19 restrictions.^65^ They have, understandably, expressed concerns and mistrust with the expectation to receive what they view as a largely experimental vaccine before the general public, particularly as other basic needs are not necessarily being met.

Communicating about the safety, efficacy, and importance of vaccination and the rationale for prioritizing these populations is a new, complex challenge for public health authorities.^66,67^ The long history of racist and unethical practice by government authorities and medical institutions in these communities has led to high levels of vaccine hesitancy. As we begin the largest vaccination campaign to date in the United States, leaders can and must show respect by recognizing the validity of vaccine hesitancy in these situations and being responsive to community concerns.

By acknowledging and acting on other concerns beyond disease control, authorities demonstrate respect—following through with concrete, responsive action is crucial for this principle. Several states and localities include small business owners and community leaders on their COVID-19 task forces and initiated targeted initiatives to support marginalized and minority communities.^68,69^ In Washington state, these commitments have been accompanied by funds to local community organizations and partners.^70^ Authorities have also incorporated messaging about where to access basic needs like food, utility and rent support, and non-COVID medical care, helping to build trust in the response and enable people to comply with other restrictions.

## Crisis Communication Lessons Learned

In addition to the 6 core communication principles outlined here and in the CERC manual, other communication lessons have been learned during the COVID-19 pandemic. Healthcare workers on the front lines have been reexamining how they communicate to patients and families. Clinicians have been clear about the need to acknowledge emotions, including fear, sadness, and anxiety, before any additional recommendations or guidance are given. Additionally, much has been learned about how to provide information to patients and their families, including the need to provide information in a concise manner free of medical terminology.^71^

The COVID-19 pandemic has severely tested the leadership and communication abilities of many individuals, including political leaders. Seen as a communication success by many is the case of New Zealand's Prime Minister Jacinda Ardern. Researchers examined speeches and public statements to identify several principles that contributed to her communication success. First, by explaining the overarching principles guiding the government response to handling COVID-19, Prime Minister Ardern conveyed proactive decisiveness and reassurance to the public that expert advice had been taken. Second, once lockdown measures were in place, she directed attention to messaging about the importance of unity and sought to reassure the public that key decisions were being driven by strong communitarian values. Third, by keeping the public aware of the overall trajectory and progress in relation to managing the COVID-19 pandemic helped ensure public acceptance of lockdown restrictions and confidence in government actions.^72^

In Vietnam, the government executed a series of information and communication campaigns to keep the public updated on the latest developments related to COVID-19. Early on in the pandemic, diverse communication channels were set up to reach the entire population. The campaign used short message service/text messaging, and music videos and short films disseminated through mass media channels and social networks such as Facebook, Zalo, YouTube, and Lotus to raise awareness. Through these different streams of communication, the campaigns quickly reached their target audiences with messages promoting awareness and behavior change actions, such as wearing masks in public areas and washing hands.^73^

## Discussion

In crises where a new threat such as COVID-19 upends the reality of the entire human population, effective communication holds tremendous potential in reducing uncertainty. Ensuring productive and effective communication is rarely easy, however, as the United States has experienced while balancing communication with politics, uncertainty, and a rapidly evolving pandemic. By examining the CERC core communication principles through examples from government and public health leaders, we can see how the United States has both excelled and failed at all levels of government and public health authority.

Despite the CERC framework's tested approach to help mitigate risk and effectively communicate to the public in an emergency, its 6 core communication principles have been applied unevenly over the pandemic thus far. This is a crucial time to review experiences at the federal, state, and local levels and gather important lessons on the effective use of CERC principles and the consequences of failing to uphold them. The importance of a unified approach to crisis communication across agencies and levels has never been clearer. A failure of administrative and public health leadership to be consistent and unified in their messaging undermines credibility, with potentially catastrophic consequences for public health and public trust—particularly as we roll out vaccines and states and localities face increasing pressures to reopen. In addition to the key communication principles outlined in this article, other critical recommendations include tailoring messaging to meet the concerns of different subpopulations, acknowledging uncertainty to express empathy and subsequently improve trust, building public trust generally to increase compliance with public health recommendations, and communicating clearly and consistently about risk so that individuals are well-equipped to take protective action.^74^

This paper has limitations. We sought to include examples that illustrated each core principle, positively and negatively. Many more communication examples could be included, but we attempted to choose examples that received national media attention. We must also acknowledge an overlap between the CERC principles, and, therefore, many examples could be used to illustrate more than 1 principle.

Despite these limitations, we believe our examination of the CERC principles within specific events during the first 12 months of the pandemic in the United States can help guide future communication efforts. States are now attempting to roll out vaccines equitably and efficiently while also managing the ongoing pandemic. To effectively tackle these new, complex crisis communication challenges, we must revisit the core principles outlined by the CDC and reframe our approach to present a unified voice, respond quickly and accurately, build credibility and public trust, and provide clear and consistent guidance, all with respect and empathy.
